# Genetic Diversity and Association Analysis for Solvent Retention Capacity in the Accessions Derived from Soft Wheat Ningmai 9

**DOI:** 10.1155/2017/2413150

**Published:** 2017-02-05

**Authors:** Peng Jiang, Ping-Ping Zhang, Xu Zhang, Hong-Xiang Ma

**Affiliations:** Provincial Key Lab for Agrobiology, Jiangsu Academy of Agricultural Sciences/Jiangsu Collaborative Innovation Center for Modern Crop Production, 50 Zhongling Street, Nanjing, Jiangsu 210014, China

## Abstract

Solvent retention capacity (SRC) test is an effective method for quality evaluation of soft wheat. Ningmai 9 is a founder in soft wheat breeding. The SRC and genotype of Ningmai 9 and its 117 derivatives were tested. Association mapping was employed to identify the quantitative trait loci (QTL) associated with SRCs. Ningmai 9 had the allele frequency of 75.60% and 67.81% to its first- and second-generation derivatives, respectively, indicating higher contribution than theoretical expectation. Neighbor-joining cluster based on the genotyping data showed that Ningmai 9 and most of its first-generation derivatives were clustered together, whereas its second-generation derivatives were found in another group. The variation coefficients of SRCs in the derivatives ranged from 5.35% to 8.63%. A total of 29 markers on 13 chromosomes of the genome were associated with the SRCs. There were 6 markers associated with more than one SRC or detected in two years. The results suggested that QTL controlling SRCs in Ningmai 9 might be different from other varieties. Markers* Xgwm44*,* Xbarc126*,* Xwmc790*, and* Xgwm232* associated with SRCs in Ningmai 9 might be used for quality improvement in soft wheat breeding.

## 1. Introduction

Soft wheat flour of low protein content is usually associated with the cookie quality [1], which produces good quality cookies with a large spread factor, such as low thickness, tender texture with smaller particle size, and low water absorption. Soft wheat yields less flour with a smaller average particle size and less damaged starch [2].

In comparing with hard wheat, solvent retention capacity (SRC) is used more often for evaluating the quality of soft wheat in cookie making [3]. SRC is the weight of solvent held by flour after centrifugation and draining. SRC tests were developed by Slade and Levine (1994) to estimate grain and end-use quality in soft wheat [4]. They are all based on a mixture of flour plus one of four different solutions: water, 5% sodium carbonate (NaCO_3_), 5% lactic acid, and 50% sucrose to predict water-holding capacity, damaged starch, gluten strength, and water soluble pentosan (arabinoxylan), respectively [3]. SRC was mainly determined by genotype [5–7]; however, most of the previous studies concerned the evaluation of SRC in different genotypes with various treatments, whereas the genetic mechanism of SRC received little attention.

Understanding genetic mechanisms and the identification of quantitative trait loci (QTL) associated with the components regulating end-use traits are the basis for quality improvement in wheat. Several mapping studies have been conducted to locate QTL associated with baking quality in wheat. However, most of them were conducted using hard wheat population. In soft wheat, Smith et al. (2011) reported large effect QTL for quality on chromosomes 1B and 2B [8]. Cabrera et al. (2015) identified 26 regions as potential QTL in a diversity panel and 74 QTL in all 5 biparental mapping populations [9].

Association mapping is a method to test the association between molecular markers and QTL based on linkage disequilibrium [10]. In recent years, it has been widely used for QTL detection in main crops, such as maize, wheat, and rice [11–13]. Generally, natural populations with wide genetic basis were used for association mapping [14, 15]. In soft wheat, Cabrera et al. (2015) identified 26 regions as potential QTL in a diversity panel from the soft wheat breeding program in USA by using an association mapping approach [9]. Zhang et al. (2015) discovered several favorable allelic variations for SRCs by association mapping with a natural population including 176 varieties (lines) from China [16].

Association mapping on founder parents and its derivatives can find some important QTL and favorable allelic variations in founder parent, which can be further used for marker assisted selection to produce more favorable varieties [17]. Ningmai 9 is a soft wheat cultivar with desirable quality and has been widely used in wheat production and as parent in the Yangtze River to Huai River valley area in China. A total of 20 cultivars derived from Ningmai 9 have been released in the past 10 years. Ningmai 9 has high general combining ability in SRCs [18]; however, the QTL and chromosome regions associated with SRCs in Ningmai 9 were unclear. In this study, the genotypes of Ningmai 9 and its derivatives were screened with SSR molecular markers covering whole-genome; meanwhile the phenotypes of SRCs were analyzed in two consecutive growth seasons. The genetic structure, genetic similarity, and association mapping were analyzed to reveal the relationship between Ningmai 9 and its derivatives and to identify molecular markers associated with SRCs.

## 2. Materials and Methods

### 2.1. Plant Materials and Phenotyping

Ningmai 9 and its 117 derivatives including 39 lines of first generation and 78 lines of second generation were used in this study (Table 1). The materials were planted in 2014 and 2015 at the experimental farm of Jiangsu Academy of Agricultural Sciences in Nanjing, China. Each line was planted in a plot comprising 3 rows with two replications. Each row was sowed with 50 seeds with the length of 1.3 m and a row-to-row distance of 0.25 m. After harvest and milling, the flour was tested for SRCs according to AACC 56-11 [19, 20]. The SRC of water, sodium carbonate, lactic acid, and sucrose were described as WSRC, SCSRC, LaSRC, and SuSRC, respectively.

### 2.2. Genotype Analysis

DNA was extracted from fresh leaves using a CTAB procedure according to Saghai-Maroof et al. (1984) [21]. One hundred and eighty-five polymorphic simple sequence repeat (SSR) primer pairs were used to screen Ningmai 9 and its 117 derived lines in the study. These markers were randomly distributed across the wheat genome, and each chromosome included 5–14 markers with an average of 8.8 markers. Map positions of markers were based on the linkage map reported by Somers et al. (2004) [22].

Each 10 *μ*L PCR contained 1 *μ*L 10 × PCR buffer, 0.6 *μ*L 15 mM MgCl_2_, 0.8 *μ*L 2 mMdNTP, 1 *μ*L 0.02 *μ*M of each primer, 0.1 *μ*LTaq DNA polymerase, 1 *μ*L 0.02 *μ*M template DNA, and 3.5 *μ*L deionized water. The cycling system consisted of an initial denaturation step of 94°C/5 min, followed by 36 cycles of 94°C/45 s, 50~60°C/45 s, 72°C/60 s, and a final extension of 72°C/10 min. Amplification bands were electrophoretically separated through nondenaturating 6% polyacrylamide gels and visualized by silver staining.

### 2.3. Data Processing and Analysis

Excel 2007 was used for data preparation; ANOVA was performed using SPSS 17.0. Neighbor-joining cluster was performed with Mega 6.0 [23]. Both the *Q* matrix and *K* matrix were determined using STRUCTURE v2.3.4 [24]. Five independent simulations were processed for each *k*, ranging from 1 to 8, with a 10,000 burn-in length and 100,000 iterations. The association analysis was calculated using the mixed linear model (MLM) method incorporated into the TASSEL 3.0 software [25]. The significant marker-trait associations were declared for *P* ≤ 0.01.

## 3. Results

### 3.1. Genetic Contribution of Ningmai 9 to Its Derivatives

A total of 490 alleles were detected with 1–7 and an average of 2.6 alleles per locus. The ratio of allele frequency between Ningmai 9 and its derivatives on the chromosomes ranged from 55.71% to 88.29% with an average of 75.60% for first generation and from 56.33% to 83.50% with an average of 67.81% for second generation (Table 2), which indicated that Ningmai 9 had a higher contribution to its derivatives than theoretically expected. Both first and second generation had highest allele frequency on chromosome 4A. The first generation possesses the higher allele frequency compared to the second on all chromosomes except for chromosome 6D.

### 3.2. Population Structure Analysis and Cluster Analysis

In order to eliminate the spurious association caused by population structure of the materials, the number of populations was calculated according to the method by Evanno et al. (2005) [26]. Two populations in the materials were previously reported in our research [27].

**Figure 1:**
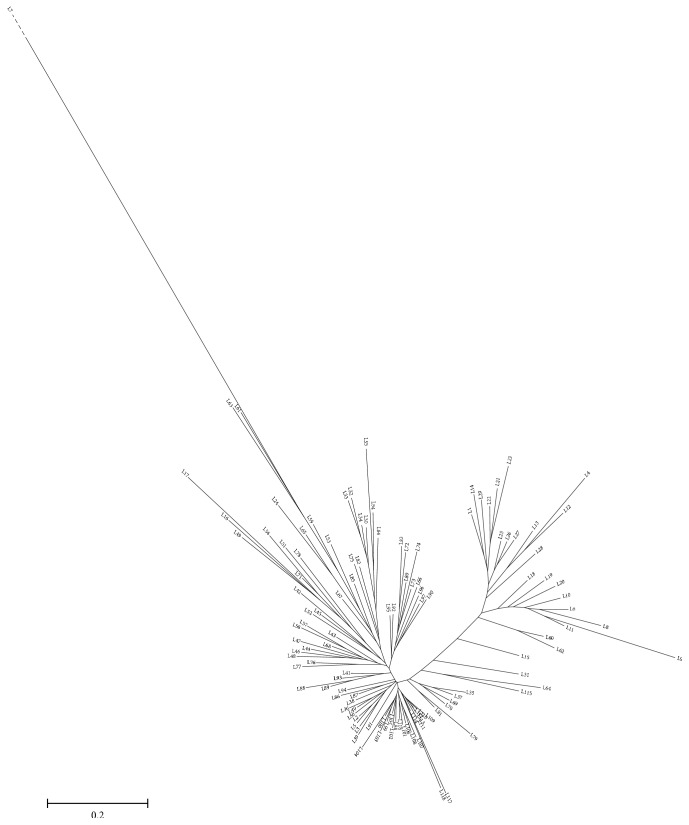
Neighbor-joining cluster of Ningmai 9 and its derivatives. Note: the genetic distance of L7 is so large that it is marked by dashed line.

Neighbor-joining cluster based on the genotyping data also showed that there were 2 groups in the materials (Figure 1). Ningmai 9 and most of its first-generation derivatives were clustered together, whereas its second-generation derivatives were found in another group. Yangfumai 4 was distantly clustered with those two groups since it was a mutant induced from hybrid seed treated with ^60^Co radiation.

### 3.3. Phenotype Analysis

There were significant variations among the derivatives of Ningmai 9 for all SRCs. The value of each SRC of the derivatives was higher, on average, than that of Ningmai 9, and the variations were high with coefficients of variation (CV) ranging from 5.35% in SuSRC (2014) to 8.63% in WSRC (2015) (Table 3).

ANOVA revealed significant effects of genotype for all SRCs (Table 4). Year effect was also significant for SCSRC. ANOVA showed that the effect of genotype by year was not significant for each SRC. There was no significant difference among generations for SRCs except for SCSRC, though the values of all the SRCs in second generation were larger than that in first generation and in Ningmai 9 except for the value of LaSRC.

There was significant positive correlation between the two years for all SRCs (Table 5). The correlation between different SRCs was identical in the two years; SuSRC was significantly positively correlated with WSRC, SCSRC, and LaSRC, and there was also significant correlation between WSRC and SCSRC.

### 3.4. Association Analysis

A total of 29 markers on 13 wheat chromosomes were associated with the SRCs (Table 6). Five markers associated with WSRC were identified on chromosomes 4A, 4D, 7B, and 7D, 21 markers on chromosomes 1B, 1D, 2A, 2B, 2D, 3B, 4A, 6A, 6B, 7A, 7B, and 7D were associated with SCSRC, two markers on chromosome 3B were associated with LaSRC, and four markers on chromosomes 1D, 2D, and 3B were associated with SuSRC. The QTL related to such markers could explain 5.12%~12.05% of the phenotypic variation.* Xgwm44* was associated with WSRC and SCSRC, and* Xwmc754* and* Xwmc326* were associated with both LaSRC and SuSRC.* Xbarc126*,* Xwmc517*,* Xgwm484*,* Xwmc754*,* Xwmc326*, and* Xgwm232* were detected in both years, and* wmc754* and* wmc326* associated with LaSRC presented different alleles in two years. Most of the alleles of the marker associated with more than one SRC or detected in two years had negative effects on their corresponding SRCs, which were a benefit for soft wheat quality.

## 4. Discussion

Ningmai 9 is a soft wheat variety with stable soft wheat quality, high yield, wide adaptation, and resistance to multiple diseases including* Fusarium* head blight, soil born mosaic virus, and sharp eye spots released in 1997. Since 2006, 20 wheat cultivars derived from Ningmai 9 have been released to wheat production of the Yangtze River to the Huai River regions in China. As a founder parent, Ningmai 9 has a high general combining ability in sterile spikelet number (negative effect), grain weight per spike, protein content (negative effect), SRC (negative effect), and* Fusarium* head blight resistance, which means that it is easy to produce desirable traits in progenies [18]. At genomic level, founder parents have more favorable allelic variations than other varieties, and the genetic composition of new varieties is more similar to founder parent rather than the average value of parents. In this study, the genetic contribution of Ningmai 9 to its first and second generation was 75.60% and 67.81%, respectively, which were both significantly higher than theoretical expectation of 50% and 25%. The result was consistent with previous reports on other founder parents, such as Triumph/Yanda 1817 [28], Orofen [29], Bima 4 [30], and Zhou 8425B [31].

Solvent retention capacity (SRC) has been considered as an important breeding tool for predicting flour functionality of different wheat for different end uses ever since it has been developed [4, 32, 33]. SRC addresses the relative contributions to water absorption of each flour component using four different solvents: water, lactic acid, sodium carbonate, and sucrose. While WSRC has been associated with the overall water-holding capacity of all flour constituents, LaSRC is associated more specifically with the glutenin network formation and gluten elasticity or strength of flour. SCSRC is closely related to the amount of damaged starch of the flour, while SuSRC relates more specifically to the concentration of arabinoxylan and gliadin [19]. In this study, SRCs of Ningmai 9 and its derivatives were measured in two consecutive years, and all the SRCs of Ningmai 9 were lower than those of the derivatives on average, as wheat breeders did not take soft wheat as the only goal in wheat breeding. Therefore, genetic improvement for soft wheat quality would be strengthened in the future.

Identification of molecular marker associated with desired traits is a basis for marker assisted selection in wheat breeding. Association mapping is an effective method for identifying related markers. In this study, a total of 29 markers on 13 chromosomes were associated with the SRCs. Five markers associated with WSRC were identified on chromosomes 4A, 4D, 7B, and 7D. Cabrera et al. [9] and Carter et al. [34] discovered QTL related to WSRC on chromosomes 4A and 4D, respectively, and the QTL on 4A was close to* Xwmc468* detected in this study. Twenty-one markers on chromosomes 1B, 1D, 2A, 2B, 2D, 3B, 4A, 6A, 6B, 7A, 7B, and 7D were associated with SCSRC in the study. Wmc751 on 3B reported by Carter et al. [34] was located at the interval between* Xwmc777* and* Xwmc653*, and* Xgwm44* on 7D was also reported by Zhang et al. [16]. Smith et al. (2011) found that a QTL on 2B associated with SCSRC was close to* Xgwm257* by using 171 families from the cross Foster/Pioneer “25R26” [8]. Some markers on chromosomes 1A, 1B, 3A, 3B, 6A, and 7A related to SCSRC were also reported [8, 9, 16], but a little far from the ones we detected, as the markers on multiple chromosomes including chromosomes 1D, 2D, and 3B associated with LaSRC and SuSRC. There was high correlation between two years for all SRCs, but only a few markers were repeatedly detected, which might be due to a limited number of markers used in this study. The association mapping in Ningmai 9 and its derivatives showed that SRCs were determined by lots of minor QTL effects but also the environment, which suggest that the genetic mechanism of SRCs was complex in Ningmai 9 and QTL controlling SRCs might differ from other varieties. The favorable allelic variations of* Xgwm44*,* Xbarc126*,* Xwmc790*, and* Xgwm232* associated with SRCs in Ningmai 9 may be used for quality improvement in soft wheat breeding.

## Figures and Tables

**Table 1:** List of Ningmai 9 and its derivatives.

Generation	Number	Variety/lines
Parent	L1	Ningmai 9

1st generation	L2	Ningmai 13
	L3	Ningmai 14
	L4	Ningmai 16
	L5	Shengxuan 6
	L6	Yangmai 18
	L7	Yangfumai 4
	L8	3E/158
	L9	Nannong 0686
	L10	Ningmai 18
	L11	Ning 0556
	L12	Ning 07123
	L13	Ning 07119
	L14	Ning 0853
	L15	Ning 0866
	L16	Ning 0894
	L17	Ning 08105
	L18	Ning 0561
	L19	Ning 0564
	L20	Ning 0565
	L21	Ning 0417
	L22	Ning 0418
	L23	Ning 0422
	L24	Ning 0311
	L25	Ning 0316
	L26	Ning 0319
	L27	Ning 0320
	L28	Ning 0327
	L29	Ning 0331
	L35	Ning 9-11
	L36	Ning 9-36
	L37	Ning 9 Large 41
	L38	Ning 9 Large 44
	L39	Ning 9 Large 76
	L40	Ning 9 Large 78
	L41	Ning 9 Large 80
	L60	71666
	L61	6E/123
	L62	09-654
	L64	09-444

	L30	Ning 0798
	L31	Ning 07117
	L32	F307
	L33	F308
	L34	Ning 0797
	L42	Ning 0862
	L43	Ning 0869
	L44	Ning 0872
	L45	Ning 0880
	L46	Ning 0882
	L47	Ning 0884
	L48	Ning 0887
	L49	Ning 0893
	L50	Ning 0895
	L51	Ning 0897
	L52	Ning 0898
	L53	Ning 0899
	L54	Ning 08102
	L55	Ning 08104
	L56	Ning 08108
	L57	Ning 08110
	L58	Ning 08115
	L59	Ning 08116
	L63	09-569
	L65	Zhenmai 166
	L66	Ning 0867
	L67	Ning 0881
	L68	Ning 0883
	L69	Ning 0886
	L70	Ning 0896
	L71	Ning 08109
	L72	Ning 08111
2nd generation	L73	Ning 08112
	L74	Ning 08113
	L75	08F331
	L76	08F333
	L77	08F337
	L78	08F353
	L79	08F362
	L80	08F386
	L81	08F387
	L82	08F396
	L83	08F397
	L84	08F399
	L85	08F406
	L86	08F407
	L87	08F408
	L88	08F409
	L89	08F410
	L90	08F411
	L91	08F417
	L92	08F418
	L93	08F423
	L94	08F424
	L95	08F426
	L96	08F432
	L97	08F433
	L98	08F434
	L99	08F435
	L100	08F436
	L101	08F437
	L102	08F442
	L103	08F443
	L104	08F444
	L105	08F445
	L106	08F446
	L107	08F448
	L108	08F449
	L109	08F450
	L110	08F451
	L111	08F453
	L112	08F454
	L113	08F457
	L114	08F458
	L115	08F468
	L116	08F459
	L117	08F516
	L118	08F517

**Table 2:** The allele frequency between Ningmai 9 and its derivatives on chromosomes.

Chromosome	Allele frequency (%)	
	1st generation	2nd generation
1A	71.28	57.33
2A	80.98	66.65
3A	71.23	64.16
4A	88.29	83.50
5A	77.09	75.34
6A	77.61	77.08
7A	74.47	60.23
Mean	77.28	69.18

1B	68.61	61.01
2B	73.08	70.61
3B	79.40	72.37
4B	76.71	64.02
5B	79.39	67.69
6B	69.97	62.60
7B	80.14	73.98
Mean	75.33	67.47

1D	75.30	66.54
2D	78.97	72.75
3D	64.86	56.33
4D	87.95	73.46
5D	72.49	64.24
6D	55.71	59.79
7D	84.09	74.27
Mean	74.19	66.77

Genome wide allele frequency with Ningmai 9 (%)		
1st generation	75.60	
2nd generation	67.81	

**Table 3:** Phenotype variation for 4 SRCs of Ningmai 9 and its derivatives.

Index	Year	Ningmai 9	Mean	Stdev	Min	Max	CV (%)
WSRC	2014	59.49	64.03	5.36	49.43	79.40	8.37
	2015	59.60	63.72	5.50	49.98	77.25	8.63
SCSRC	2014	78.96	86.73	6.86	71.11	98.91	7.91
	2015	79.85	85.66	6.97	70.38	100.15	8.13
LaSRC	2014	108.52	116.88	9.24	93.98	141.71	7.91
	2015	109.05	117.23	9.57	96.21	146.57	8.16
SuSRC	2014	108.85	116.05	5.97	99.15	131.09	5.15
	2015	109.51	117.06	6.45	104.39	132.75	5.51

**Table 4:** ANOVA and multiple comparisons among generations for the SRCs of Ningmai 9 and its derivatives.

Index	*F* value			Multiple comparison test (S-N-K method)		
	Genotype	Year	Genotype × year	Ningmai 9	1st generation	2nd generation
WSRC	5.24^*∗∗*^	0.52	0.28	59.55^a^	61.15^a^	65.29^a^
SCSRC	8.04^*∗∗*^	6.02^*∗*^	0.77	79.41^a^	81.14^ab^	88.81^b^
LaSRC	4.75^*∗∗*^	0.21	0.54	108.79^a^	117.72^a^	116.83^a^
SuSRC	3.43^*∗∗*^	3.00	0.51	109.18^a^	115.66^a^	117.10^a^

*∗∗* and *∗* show significant difference at 0.01 and 0.5 level, respectively; different small letters in the same row show significant difference at 0.05 level.

**Table 5:** Correlation analysis for the SRCs over two years in Ningmai 9 and its derivatives.

	WSRC	SCSRC	LaSRC	SuSRC
WSRC	0.897^*∗∗*^	0.663^*∗∗*^	−0.012	0.343^*∗∗*^
SCSRC	0.640^*∗∗*^	0.825^*∗∗*^	−0.007	0.525^*∗∗*^
LaSRC	0.037	0.085	0.809^*∗∗*^	0.403^*∗∗*^
SuSRC	0.311^*∗∗*^	0.582^*∗∗*^	0.398^*∗∗*^	0.740^*∗∗*^

*∗∗* shows significant difference at 0.01 level. The correlation analysis for the same trait between 2014 and 2015 is marked on the diagonal; the correlation analysis among different traits in 2014 is marked below the diagonal, whereas the correlation analysis among different traits in 2015 is marked above.

**Table 6:** Association analysis for SRCs.

Traits	Marker	Chromosome	2014			2015		
			*P*	*R* ^2^ (%)	Effect (allele)	*P*	*R* ^2^ (%)	Effect (allele)
WSRC	*Xwmc468*	4AL	4.74 × 10^−3^	6.61	− (134)			
	*Xwmc89*	4DS	8.10 × 10^−3^	5.94	− (140)			
	*Xwmc517*	7BL				9.68 × 10^−3^	5.71	+ (183)
	*Xgwm44*	7DS	8.11 × 10^−3^	5.78	− (196)	1.37 × 10^−3^	8.84	− (196)
	*Xbarc126*	7DS	6.48 × 10^−3^	6.13	− (170)	4.34 × 10^−4^	10.80	− (170)

SCSRC	*Xgwm153*	1BL				5.65 × 10^−3^	6.02	− (188)
	*Xcfd72*	1DL				4.55 × 10^−3^	6.22	+ (310)
	*Xgwm232*	1DL	7.64 × 10^−4^	8.57	+ (144)			
	*Xwmc658*	2AL	6.82 × 10^−3^	5.44	+ (250)			
	*Xgwm257*	2BS				9.54 × 10^−3^	5.16	− (186)
	*Xgwm539*	2DL	6.32 × 10^−3^	5.56	+ (160)			
	*Xgwm102*	2DS	3.04 × 10^−3^	6.57	+ (142)			
	*Xgwm484*	2DS	1.94 × 10^−4^	10.81	− (179)	2.90 × 10^−3^	6.96	− (179)
	*Xwmc231*	3B	1.09 × 10^−3^	8.17	+ (240)			
	*Xwmc777*	3B	4.13 × 10^−4^	9.48	− (100)			
	*Xwmc653*	3B				6.05 × 10^−3^	5.83	− (160)
	*Xwmc219*	4AL	6.68 × 10^−3^	5.47	+ (160)			
	*Xgwm169*	6AL				9.54 × 10^−3^	5.68	− (190)
	*Xwmc397*	6BL	9.68 × 10^−5^	12.05	—			
	*Xwmc790*	7A	1.68 × 10^−3^	7.42	− (108)			
	*Xwmc809*	7A				6.04 × 10^−3^	5.81	− (180)
	*Xwmc311*	7BL	9.64 × 10^−3^	5.12	+ (120)			
	*Xwmc634*	7DL	4.16 × 10^−4^	10.17	+ (210)			
	*Xgwm437*	7DL				6.04 × 10^−3^	5.81	− (110)
	*Xgwm44*	7DS				6.61 × 10^−3^	5.88	− (183)
	*Xcfd14*	7DS				1.80 × 10^−3^	7.62	− (100)

LaSRC	*Xwmc754*	3B	2.09 × 10^−3^	8.77	− (160)	7.89 × 10^−3^	6.39	+ (152)
	*Xwmc326*	3B	7.20 × 10^−3^	7.15	+ (186)	8.29 × 10^−3^	7.00	+ (186)

SuSRC	*Xgwm232*	1DL	5.17 × 10^−4^	10.44	− (144)	4.27 × 10^−3^	7.20	− (144)
	*Xgwm349*	2DL	5.97 × 10^−3^	6.47	+ (310)			
	*Xwmc754*	3B	5.96 × 10^−3^	6.68	− (160)			
	*Xwmc326*	3B				4.38 × 10^−3^	7.35	+ (186)

The number in brackets following “+” or “−” represents the allele of markers, and “+” and “−” indicate a positive or negative effect by the allele of markers.
